# Metabolic correlates of late midlife cognitive outcomes: findings from the 1946 British Birth Cohort

**DOI:** 10.1093/braincomms/fcab291

**Published:** 2021-12-15

**Authors:** Rebecca Green, Jodie Lord, Jin Xu, Jane Maddock, Min Kim, Richard Dobson, Cristina Legido-Quigley, Andrew Wong, Marcus Richards, Petroula Proitsi

**Affiliations:** Institute of Psychiatry, Psychology & Neuroscience, King’s College London, London, UK; UK National Institute for Health Research (NIHR) Maudsley Biomedical Research Centre, South London and Maudsley Trust, London, UK; Institute of Psychiatry, Psychology & Neuroscience, King’s College London, London, UK; Institute of Psychiatry, Psychology & Neuroscience, King’s College London, London, UK; Institute of Pharmaceutical Science, King’s College London, London, UK; MRC Unit for Lifelong Health & Ageing at UCL, University College London, London, UK; Steno Diabetes Center Copenhagen, Gentofte, Denmark; Institute of Psychiatry, Psychology & Neuroscience, King’s College London, London, UK; UK National Institute for Health Research (NIHR) Maudsley Biomedical Research Centre, South London and Maudsley Trust, London, UK; Health Data Research UK London, University College London, London, UK; NIHR Biomedical Research Centre at University College London, Hospitals NHS Foundation Trust, London, UK; Institute of Pharmaceutical Science, King’s College London, London, UK; Steno Diabetes Center Copenhagen, Gentofte, Denmark; MRC Unit for Lifelong Health & Ageing at UCL, University College London, London, UK; MRC Unit for Lifelong Health & Ageing at UCL, University College London, London, UK; Institute of Psychiatry, Psychology & Neuroscience, King’s College London, London, UK

**Keywords:** metabolomics, dementia, cognition, epidemiology, network analysis

## Abstract

Investigating associations between metabolites and late midlife cognitive function could reveal potential markers and mechanisms relevant to early dementia. Here, we systematically explored the metabolic correlates of cognitive outcomes measured across the seventh decade of life, while untangling influencing life course factors. Using levels of 1019 metabolites profiled by liquid chromatography–mass spectrometry (age 60–64), we evaluated relationships between metabolites and cognitive outcomes in the British 1946 Birth Cohort (*N* = 1740). We additionally conducted pathway and network analyses to allow for greater insight into potential mechanisms, and sequentially adjusted for life course factors across four models, including sex and blood collection (Model 1), Model 1 + body mass index and lipid medication (Model 2), Model 2 + social factors and childhood cognition (Model 3) and Model 3 + lifestyle influences (Model 4). After adjusting for multiple tests, 155 metabolites, 10 pathways and 5 network modules were associated with cognitive outcomes. Of the 155, 35 metabolites were highly connected in their network module (termed ‘hub’ metabolites), presenting as promising marker candidates. Notably, we report relationships between a module comprised of acylcarnitines and processing speed which remained robust to life course adjustment, revealing palmitoylcarnitine (C16) as a hub (Model 4: *β* = −0.10, 95% confidence interval = −0.15 to −0.052, *P* = 5.99 × 10^−5^). Most associations were sensitive to adjustment for social factors and childhood cognition; in the final model, four metabolites remained after multiple testing correction, and 80 at *P* < 0.05. Two modules demonstrated associations that were partly or largely attenuated by life course factors: one enriched in modified nucleosides and amino acids (overall attenuation = 39.2–55.5%), and another in vitamin A and C metabolites (overall attenuation = 68.6–92.6%). Our other findings, including a module enriched in sphingolipid pathways, were entirely explained by life course factors, particularly childhood cognition and education. Using a large birth cohort study with information across the life course, we highlighted potential metabolic mechanisms associated with cognitive function in late midlife, suggesting marker candidates and life course relationships for further study.

## Teaser

Green *et al*. identify metabolic mechanisms underlying cognitive function in late midlife, highlighting key metabolites that may present as markers of cognitive health and dementia risk. The authors further identify influencing life course factors, including a large impact of childhood cognition and education on many associations.

## Graphical Abstract

**Graphical Abstract:**
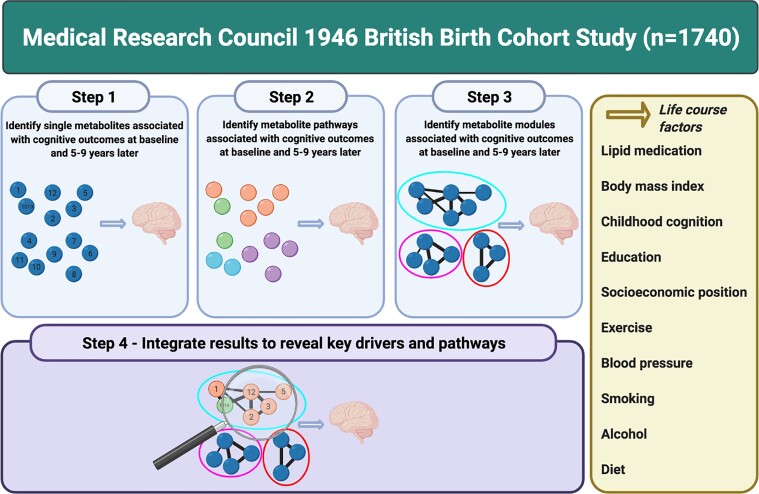

## Introduction

Cognitive function in the seventh decade of life is indicative of future cognitive trajectories and risk of dementia.^1^ As dementia is proposed to have a long prodrome, where pathology is accumulating but clinical criteria are not yet met, there presents a promising window to prevent or delay pathology.^1^ However, a lack of clinically significant symptoms impedes our ability to identify individuals for potential risk reduction and treatment strategies. As such, our understanding of early disease mechanisms are not well established and no effective disease-modifying treatments are currently used in the clinic.^2^

Comprehensive longitudinal studies are required to detect early mechanisms and markers preceding diagnosis, for which studying metabolic correlates may be fruitful. Metabolites, such as fatty acids and amino acids, are low molecular weight compounds derived from cellular metabolism. Lying in closest proximity to the phenotype, they can reflect upstream biological systems (e.g. genetics, transcriptomics, proteomics) as well as environmental and lifestyle influences, allowing for a holistic insight into the physiological status of an individual.^3^ Additionally, they are accessible and potentially modifiable, and thus present as promising targets of intervention.^4^

The biological relevance of metabolic alterations in cognitive function and dementia has been established. Contextually, genome-wide association studies (GWAS) have highlighted enrichment in lipid metabolism pathways in the genetic underpinnings of Alzheimer's disease.^5^ Further, many studies have linked metabolites to cognitive function and Alzheimer's disease, consistently highlighting species such as sphingolipids, phospholipids, fatty acids, cholesterol and amino acids,^6–11^ although replication of specific metabolites has proved challenging. Inconsistencies in accounting for lifestyle and environmental influences, potential confounding by reverse causation and heterogeneity in clinical states have been suggested to contribute to replication issues.^8^ Genetic approaches such as Mendelian randomization (MR) have provided further insights into causality^12^ but require well-powered GWAS in order to avoid weak instrument bias, which are not always available.

Looking at associations of single metabolites can allow for granular insights into the molecular correlates of cognitive outcomes. However, metabolites are highly correlated, and biological processes are likely to involve a coordinated effort of many metabolites. Systems-level analyses, which are able to capture the complex interactions between metabolites, are required to guide our understanding and identify potential marker candidates for future studies. One such approach is weighted gene correlation network analysis (WGCNA), which can be applied to other -omics modalities, whereby data are organized into modules based on pairwise correlations.^3–5^ Relationships between modules and outcomes can be subsequently explored, and metabolites that are highly connected in their module (hereby named ‘hub’ metabolites) can be identified. As these metabolites are influential in module structure and likely to play key roles in biological function, they present as promising marker candidates for further study.

A number of studies, including ours, have applied WGCNA to identify molecular profiles associated with Alzheimer's disease and Alzheimer's disease endophenotypes.^6–8^ However, studies linking metabolites to cognitive outcomes have typically been directed towards clinical phenotypes where irreversible damage has already occurred. To our knowledge, the molecular correlates of cognitive function relevant to this prodromal window are yet to be explored using a systems approach, and the influence of life course factors has not been previously considered. It is hoped that this could highlight independent associations as well as suggest relationships for further study—a potentially invaluable layer in untangling early pathology.

Using the Medical Research Council (MRC) National Survey of Health and Development (NSHD)—the British 1946 Birth Cohort—we aimed to comprehensively investigate associations between metabolites and cognitive function at ages 60–69 using a life course approach (see Graphical Abstract). Integrating the depth and breadth of metabolite-level, pathway-level and network-level approaches, we aimed to identify metabolites that may show merit as markers of early pathology. With lifelong information available, we explored the influence of life course factors to untangle these associations further.

## Materials and methods

### Participants

The MRC NSHD originally consisted of 5362 participants born in mainland Britain during 1 week of March in 1946.^19^ Twenty-four waves of data have been collected since birth, with the most recent follow-ups at ages 60–64 (*n* = 2229) and 68–69 (*n* = 2148). The study sample remains broadly representative of the British-born population at the same age.^19,20^ Participants with full cognition, metabolite and blood clinic data at age 60–64 were included for this analysis (*N* = 1740, 50.9% female). Blood samples and age 60–64 cognitive measures were collected during the same clinic visit.

Ethical approval was obtained from the Multicentre Research Ethics Committee (for data collections up to 2010), and the Scotland A Research Ethics Committee (14/SS/1009) and Queen Square Research Ethics Committee (13/LO/1073) (for data collections between 2014 and 2015). Research was conducted in accordance with the Declaration of Helsinki and participants provided written informed consent at each wave.

### Metabolomics

Blood samples were collected by a research nurse at age 60–64 (96% fasted). Samples were aliquoted and stored at −80°C.

Levels of 1401 plasma metabolites were profiled by *Metabolon* (Durham, NC, USA) using Ultrahigh Performance Liquid Chromatography-Tandem Mass Spectrometry (UPLC-MS/MS) (Supplementary Methods). Metabolites were assigned to nine families and further organized into pathways by *Metabolon* based on their proposed biological function informed by the KEGG (Kyoto Encyclopaedia of Genes and Genomes) database (see Supplementary Table 1). The identity of 291 metabolites was unknown; these metabolites were allocated numbers prefixed by an ‘X’ and were not assigned to any family.

Our data quality control pipeline is presented in Supplementary Figure 1. Briefly, metabolites with >20% of missing data were excluded, leaving 1019 for further analysis. The remaining missing data were then imputed using *k*-nearest-neighbours (*k* = 10), as recommended elsewhere.^21^ Imputed data were then log10 transformed to achieve approximately normal distributions.

### Cognitive outcomes

Cognitive outcome measures were recorded at ages 60–64 and 69. Four aspects of cognitive function were assessed.

#### Short-term memory (ages 60–64 and 69)

Participants were asked to recall a 15-item word list, developed by the NSHD, after being presented with each word for 2 s. The task was repeated over three trials and the number of accurately recalled words was recorded (max score = 45).^22^

#### Processing speed (ages 60–64 and 69)

Participants were asked to cross out the letters P and W, randomly distributed on a page containing other letters. One minute was given to complete the task and participants were scored by the number and accuracy of the letters crossed out (max score = 600).^22^

#### Delayed memory (age 60–64)

After the processing speed task, participants were asked to recall the 15-item word list presented for the short-term memory measure (max score = 15).^22^

#### Addenbrooke's cognitive examination-III (age 69)

The Addenbrooke’s Cognitive Examination-III (ACE-III) captures cognitive state, and is also a screening tool for cognitive impairment, comprised of five domains: attention and orientation, verbal fluency, memory, language and visuospatial function. Scores represent the total over all domains (max score = 100), with lower scores indicating poorer cognitive function.^23^

#### Cognitive change

For outcomes with available data at two time points—short-term memory and processing speed—we additionally investigated change in cognition, represented by the standardized residuals of a regression model fit between scores at ages 60–64 and 69.

### Covariables

As with previous analyses,^6^ covariables included the following: sex, blood clinic information (age at blood collection, clinic location and fasting status), body mass index (BMI), lipid medication, childhood cognition, educational attainment, childhood socioeconomic position (SEP), midlife SEP, lifetime smoking, alcohol intake, systolic blood pressure, physical activity and diet.

*BMI* was calculated using height and weight measures collected during the nurse visit at ages 60–64. At blood collection, the self-reported use of *lipid medication* was recorded and coded as a binary variable reflecting use in the previous 24 h.

*Childhood cognition* was represented as a standardized composite score of four tests at age 15, including the Heim AH4 (measuring non-verbal and verbal ability),^24^ the Watts Vernon reading test (measuring reading comprehension)^25^ and a test of mathematical ability.^25^  *Educational attainment* represented the highest level of educational qualification by age 26, grouped into three categories: no qualifications, ordinary (‘O’) level secondary qualifications or advanced (‘A’) level secondary and higher. *SEP* was represented in childhood and midlife, coded using the current or last known occupation of the father at age 11 and the study member at age 53, respectively. These categories corresponded to those specified in the UK Registrar General’s classification: unskilled, partly skilled, skilled manual, skilled non-manual, intermediate or professional.

For lifestyle, *lifetime smoking* was represented by pack years per person between the ages of 20 and 60–64. *Physical activity* was coded as three categories depending on the self-reported frequency of participation in sports, exercises or intense leisure activities in the month prior to the age 60–64 interview: none, 1–4 times per week or >4 times per week. Where data were present for at least three of four timepoints, *average daily alcohol intake* during midlife was curated from 3 to 5 day diet diaries at ages 36, 43, 53 and 60–64. This measure was then used to assign participants into three categories: no consumption (0 units per day), light-to-moderate consumption (females: <3 units per day, males: <4 units per day) and heavy consumption (females: >3 units per day, males: >4 units per day). *Systolic blood pressure* was represented by the second measurement (mmHg) taken at age 60–64. Finally, the *diet* variable represented adherence scores for the Dietary Approaches to Stop Hypertension (DASH) diet, estimated from 3 to 5 day diet diaries at age 60–64.^26^ The DASH diet is based on a high intake of fruits, vegetables, low-fat dairy products and wholegrains, and a low intake of saturated fat and refined sugars.^27^ Participants were assigned to sex-specific quintiles, with lower quintiles indicating lower adherence, as described previously.^26^

*Apolipoprotein E* (*APOE)* genotype was determined from blood samples collected at age 53 or 69 and analysed as described previously.^28^ The proportion of *APOE* genotypes represented among participants is described in Supplementary Table 2. Due to potentially opposing effects of ε2 and ε4 alleles on dementia risk, participants with ε2/ε4 were excluded (*n* = 48) and *APOE* genotype was coded as homozygous ε4 (*n* = 46), heterozygous ε4 (*n* = 361) or non ε4 (*n* = 1068). Genotypes were treated as continuous variables.

### Statistical analyses

Analyses were carried out in R version 3.6.0 (packages: impute,^29^ WGCNA,^14,15^ mice,^30^ VIM,^31^ dplyr,^32^ ggplot2,^33^ ComplexUpset,^34^ gridExtra^35^). Missing covariable data were imputed using multiple imputation chained equations, resulting in 50 imputed data sets. Prior to statistical analysis, all predictors and outcomes were standardized to a mean of 0 and standard deviation of 1 to allow for direct comparisons.

For all regression analyses, assumptions of linearity were checked by examination of the residuals. Due to high intercorrelation between cognitive outcomes, Bonferroni corrections were applied to the metabolite data only, to control for the number of independent metabolites and hence the number of independent tests performed.

### Single-metabolite analyses

Associations between metabolites and cognitive outcomes were evaluated using multiple linear regression. Regression analyses were performed on each imputed data set, and estimates were pooled using Rubin's rules. To investigate associations in the context of life course influences, a series of statistical models were performed:

1. Model 1 (basic covariables): sex, blood clinic, age at blood clinic, fasting status;
2. Model 2 (common metabolite confounders): Model 1 + BMI, lipid medication;
3. Model 3 (social factors and childhood cognition): Model 2 + childhood cognition, attainment, SEP (childhood and midlife);
4. Model 4 (lifestyle influences): Model 3 + blood pressure, physical activity, alcohol, smoking, diet.

As liquid chromatography–mass spectrometry (LC–MS) is highly sensitive and able to capture different conformations of the same metabolite species (correlations between metabolites in the full data set ranged from −0.68 to 0.998), we applied a multiple testing correction using an approach applied previously.^6^ A Bonferroni-adjusted significance threshold was set to *P* < 1.15 × 10^−4^; 0.05/435 (the number of principal components explaining >95% variance in the 1019 metabolites).

### Pathway analyses

Metabolites were assigned to pathways based on Metabolon pathway definitions. Those containing <5 metabolites were excluded, resulting in 53 pathways.

Quantitative pathway analyses were performed using an approach reported previously.^36^ Briefly, we derived pathway scores for each participant, representing the mean standardized expression of metabolites in the pathway. To do this, metabolites were *z*-standardized, and the mean expression was computed for each pathway. Associations between pathways and outcomes were evaluated using linear regression, adjusting for the basic (Model 1) covariables listed above. A Bonferroni-corrected significance threshold was set at 0.05/53 pathways; *P* < 9.43 × 10^−4^.

### Network analyses

#### Network construction

To define metabolic networks, we applied WGCNA to metabolite data.^3–5^ Metabolites were first adjusted for Model 1 covariables and the standardized residuals were used for subsequent analysis. Next, the standardized connectivity (*Z*.*k*) for each sample was computed to identify outliers, resulting in the exclusion of 10 individuals with a *Z*.*k* of < −4. We then derived a pairwise correlation matrix using biweight midcorrelations between all metabolites. From this, a weighted, signed adjacency matrix was constructed by raising correlations to a soft thresholding power of 9, chosen to meet a scale-free topology threshold of ≥0.85 while maximizing mean connectivity (Supplementary Figure 2). Subsequently, the adjacency matrix was transformed into a topological overlap matrix (TOM), representing the network connectivity of metabolites. Metabolites were then hierarchically clustered into a dendrogram using an average linkage method based on their dissimilarity (1−TOM), and the dendrogram was cut using a dynamic hybrid tree cutting algorithm^37^ [parameters—minModuleSize = 20 (default), deepSplit = 4 (to allow for more granular modules) and mergeHeight = 0.25 (default)], resulting in 15 metabolite modules. Of these, the ‘grey’ module, comprised of metabolites that were not assigned to any particular module, was dropped from further analysis. Module eigenvalues were computed for the remaining 14 modules.

Overrepresentation analyses using the hypergeometric test were performed on modules to identify pathways expressed more than expected by chance. For all module analyses, a Bonferroni-corrected significance threshold was set at 0.05/14 modules; *P* < 3.57 × 10^−3^.

#### Regression analyses

Modules were subject to the same series of regression models listed in 2.5.1, using module eigenvalues as predictors. As modules were adjusted for Model 1 covariables, these were not additionally included.

#### Module hubs

To identify hubs, we evaluated associations between metabolites and their assigned module (module membership; kME) using correlations between metabolites and module eigenvalues. Metabolites with a kME > 0.65 were defined as hubs, and additionally filtered for those identified in single-metabolite analyses.

### Additional analyses

In our preliminary analysis, we investigated associations between all covariables and metabolites, and all covariables and outcomes (adjusting for Model 1 covariables) (Supplementary Table 3). To further investigate whether particular covariables may be driving attenuations, we repeated single-metabolite and module regression analyses, adjusting for Model 1 covariables and each additional covariable individually (Supplementary Tables 4 and 5).

For significant results, analyses were rerun additionally adjusting for *APOE* genotype to investigate whether relationships were independent of *APOE* (Supplementary Table 6). We additionally repeated our analyses excluding participants below the clinical threshold (<82/100) for ACE-III^22^ (*n* = 65) (Supplementary Table 7).

### Data availability

Data used in this publication are available to researchers upon request, review and approval by the NSHD Data Sharing Committee. Further details can be found at https://www.nshd.mrc.ac.uk/data/data-sharing/.

## Results

### Participant characteristics

Complete metabolite, cognition and blood clinic data at age 60–64 were available for 1740 study participants. Repeated measures at age 69 were present for 1482 (short-term memory) and 1496 (processing speed), and ACE-III scores were present for 1255. Participant characteristics are shown in Supplementary Table 2.

### Single-metabolite analyses

Overall, we identified 155 metabolites to be associated with a least one cognitive outcome after adjusting for multiple tests (Supplementary Figure 3; see Supplementary Table 1 and Supplementary Figure 4 for full summary data). Correlations between the 155 metabolites ranged from −0.40 to 0.93. No metabolites were associated with cognitive change.

The bulk of associations attenuated after adjusting for social factors and childhood cognition (Model 3), with seven metabolites remaining significant at the adjusted threshold. In the final model, four of these remained [X—17676, palmitoylcarnitine (C16), margaroylcarnitine (C17)* and imidazole propionate; see Table 1], and 80 metabolites were nominally significant.

**Table 1:** Biological family, pathway and key results for significant (*P* < 1.15 × 10^−4^) metabolites after adjusting for life course factors (Model 4)

Metabolite	Family	Pathway	Module (kME)	Model 1			Model 4			*β* change Model 1–4 (%)
				*β*	95% CI	*P*-value	*β*	95% CI	*P*-value	
X—17676	Unknown	Unknown	Turquoise (0.52)	STM (69 years)						
				**−**0.14	**−**0.19 to −0.089	5.29 × 10^−8^	−0.11	−0.15 to −0.06	4.84 × 10^−6^	−24.69%
				DM (60–64 years)						
				−0.11	**−**0.15 to −0.061	5.45 × 10^−6^	−0.082	−0.12 to −0.04	1.11 × 10^−4^	−23.56%
Palmitoylcarnitine (C16)	Lipid	Fatty acid metabolism (Acyl Carnitine)	Purple (0.73)	PS (60–64 years)						
				−0.11	−0.16 to −0.062	9.19 × 10^−6^	−0.10	−0.15 to −0.052	5.99 × 10^−5^	−9.49%
Margaroylcarnitine (C17)*	Lipid	Fatty acid metabolism (Acyl Carnitine)	Yellow (0.46)	PS (60–64 years)						
				−0.067	−0.11 to 0.020	5.40 × 10^−3^	−0.095	−0.14 to −0.048	−8.33 × 10^−5^	+40.75%
Imidazole propionate	Amino acid	Histidine metabolism	Turquoise (0.37)	STM (69 years)						
				−0.14	−0.19 to −0.091	4.98 × 10^−8^	−0.094	−0.14 to −0.048	7.91 × 10^−5^	−33.48%

STM, short-term memory; DM, delayed memory; PS,  processing speed; KME, module membership.

### Pathway analyses

**Figure 1:**
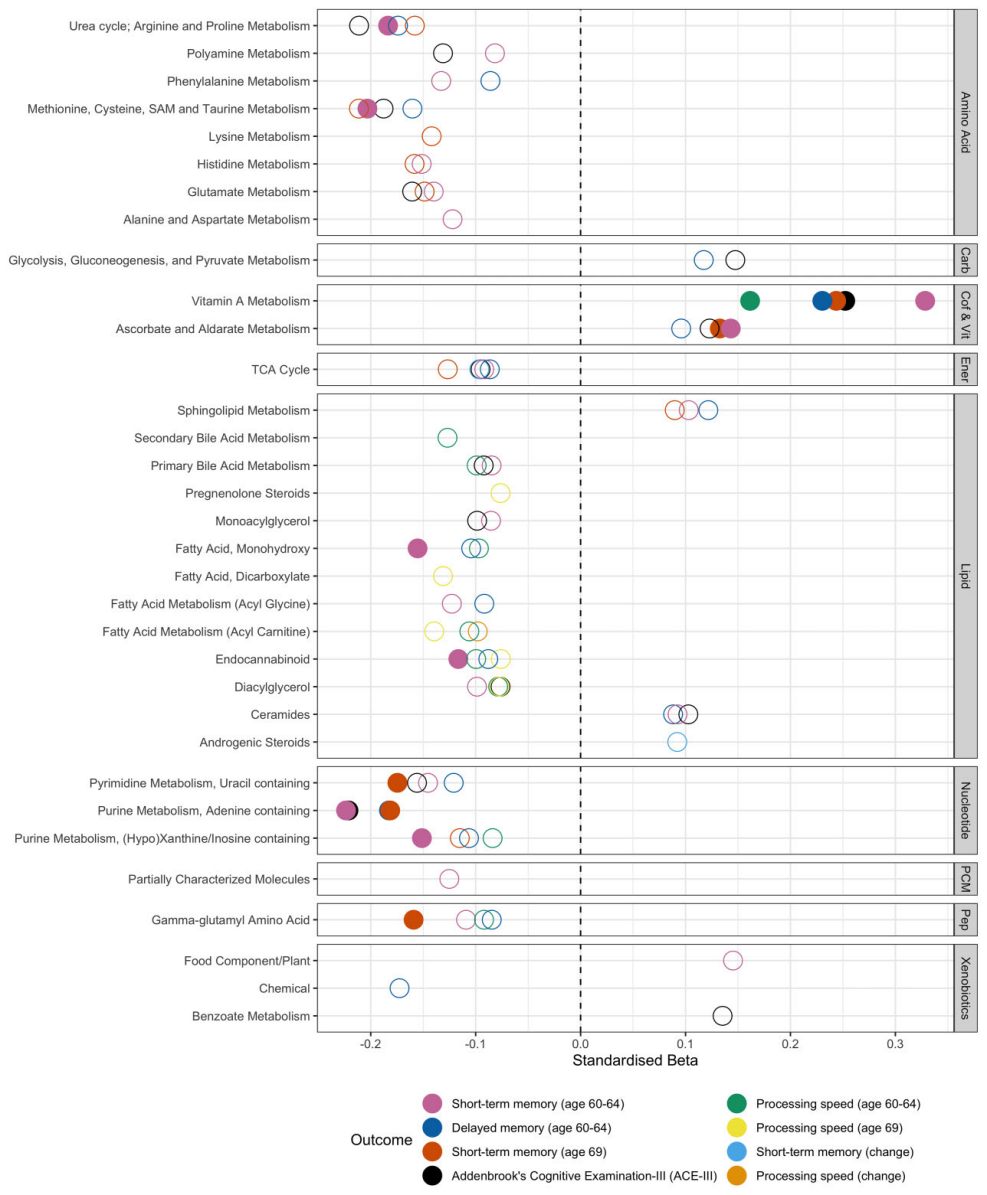
**Plot representing pathway–outcome associations, organized by metabolite family**. Bonferroni-significant pathways (*P* < 9.43 × 10^−4^) are represented by a solid fill and nominal metabolites by no fill (*P* < 0.05). Source data are present in Supplementary Table 8. Carb, carbohydrates; Cof & Vit, cofactors and vitamins; Ener, Energy; PCM, partially characterized molecules; Pep, peptides.

Results of our pathway analyses are presented in Fig. 1 and Supplementary Table 8. After adjusting for multiple tests, 10 pathways showed associations with cognitive outcomes. No pathway was significant for processing speed (69 years), nor for cognitive change (*P* > 9.43 × 10^−4^). For all other outcomes, positive relationships were seen for the Vitamin A metabolism pathway, as well as the ascorbate and aldarate metabolism pathway and short-term memory at both time points.

We observed negative relationships between the purine metabolism (adenine containing) pathway and the ACE-III and memory outcomes (short-term and delayed). Associations were additionally seen between various pathways belonging to amino acid, lipid, nucleotide and peptide families and short-term memory at each time point, although these pathways were nominally associated with other outcomes (Fig. 1).

### Network analyses

**Figure 2:**
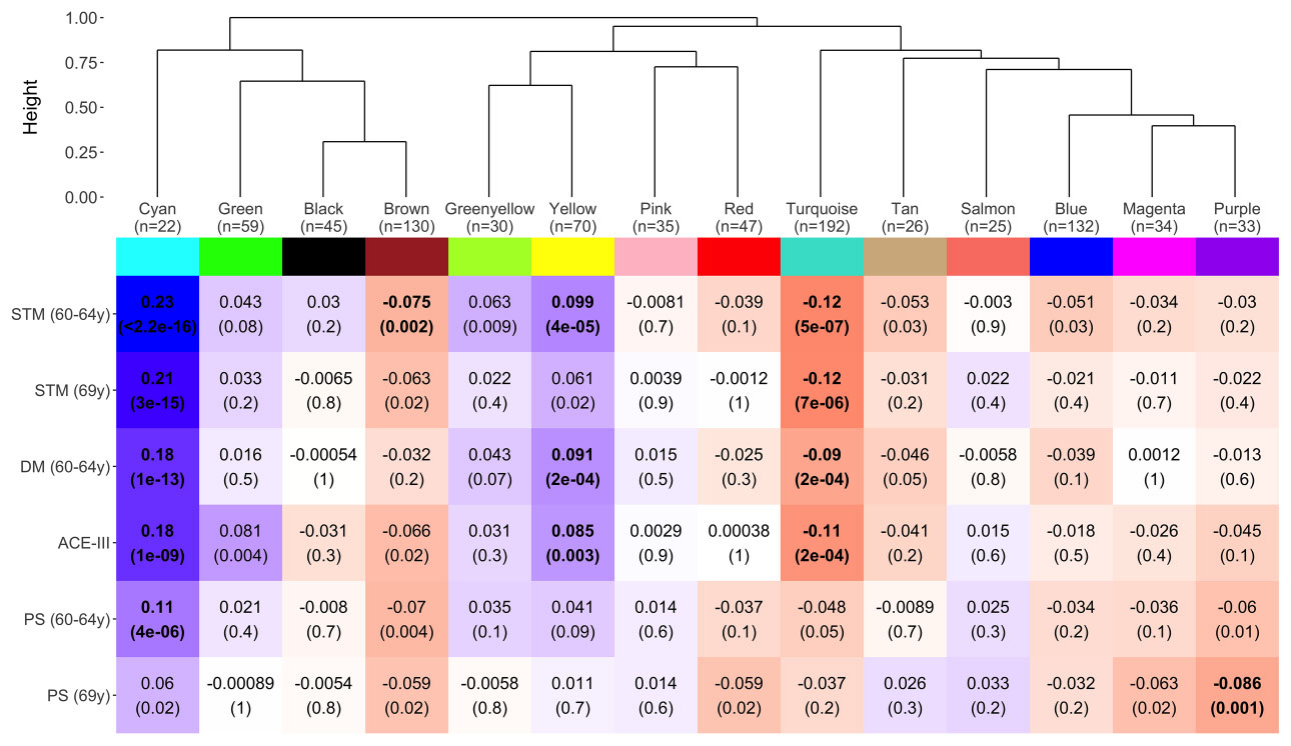
**Module dendrogram and heatmap of module–outcome associations (basic model)**. Effect sizes and unadjusted *P*-values are presented in the tiles and significant associations (*P* < 3.57 × 10^−3^) are highlighted in bold. For clarity purposes, only outcomes demonstrating a Bonferroni-significant result are shown. STM, short-term memory; DM, delayed memory; ACE-III, Addenbrooke’s Cognitive Examination-III; PS, processing speed.

**Figure 3:**
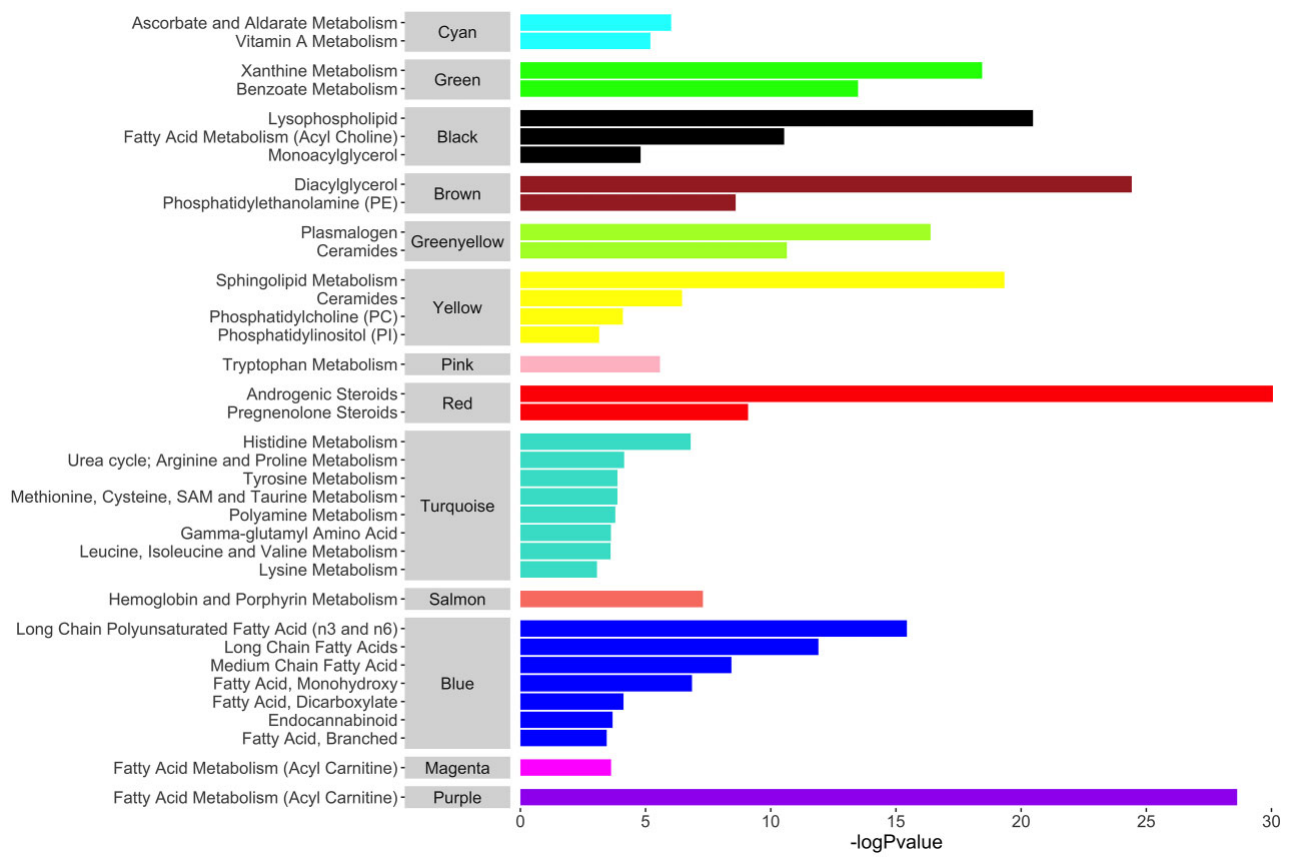
**Overrepresented pathways in network modules**. Only pathways significant at the adjusted threshold (*P* < 9.43 × 10^−4^) are shown. *P*-values are unadjusted. Source data are present in Supplementary Tables 9 and 10.

**Figure 4:**
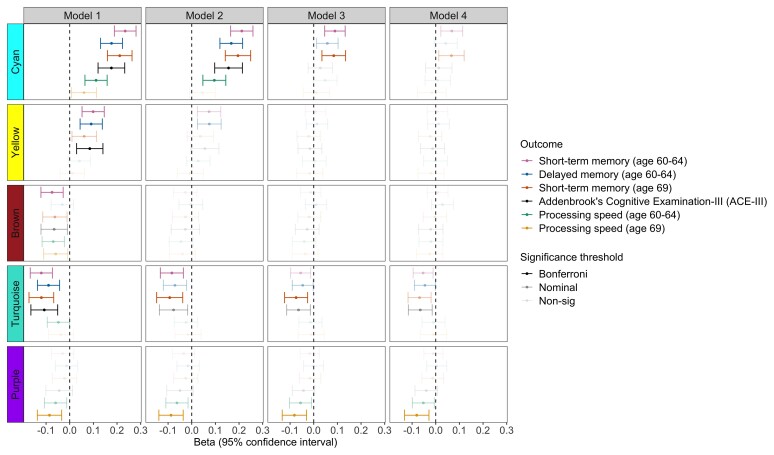
**Forest plot showing associations between modules and outcomes in Models 1–4**. Bonferroni-significant modules (*P* < 3.57 × 10^−3^) are represented by a solid fill, nominal modules (*P* < 0.05) by a fainter fill and modules that are not significant at either threshold are represented by the faintest fill. For clarity purposes, only outcomes demonstrating a Bonferroni-significant result are shown. Source data are present in Supplementary Table 10.

WGCNA analysis identified 14 modules comprising 22–192 metabolites; five of these were associated with cognitive outcomes in Model 1 at the adjusted threshold (*P* < 3.57 × 10^−3^) (Fig. 2). All but one module were enriched in a biological pathway (Fig. 3 and Supplementary Table 9), and no relationships were seen for cognitive change measures (*P* > 3.57 × 10^−3^). Key results are presented in Figs 2–4 and summary statistics in Supplementary Table 10.

#### Positive relationships (cyan and yellow)

After adjusting for multiple tests, the cyan module—enriched in ascorbate and aldarate metabolism, vitamin A metabolism and food/plant consumption—was associated with all memory outcomes, processing speed (64 years) and ACE-III (Model 1: *β* range = 0.11– 0.23, *P* range ≤ 2.16 × 10^−16^ to 3.52 × 10^−6^). In the final model, no relationships were significant at the adjusted threshold, but associations for short-term memory remained nominally associated (60–64 years: *P* = 4.10 × 10^−3^; 69 years: *P* = 0.015). Overall attenuations ranged from 68.6 to 92.6%.

We similarly found the yellow module—enriched in sphingolipid metabolism and related pathways—to display positive associations with ACE-III and age 60–64 memory outcomes at the adjusted threshold (Model 1: *β* range = 0.085 to 0.10, *P* range = 3.64 × 10^−5^ to 2.85 × 10^−3^). These findings were substantially or fully attenuated in the final model (87.0–116%).

For both the cyan and yellow modules, relationships were most sensitive to Model 3 adjustments, with childhood cognition and education resulting in the biggest attenuations (Supplementary Table 5).

#### Negative relationships (turquoise, purple and brown)

Negative associations were identified between the purple module—enriched in fatty acid (acyl carnitine) metabolism—and processing speed (age 69) [Model 1: *β* = −0.086, 95% confidence interval (CI) = −0.14 to −0.034, *P* = 1.15 × 10^−3^]. This relationship remained significant at the adjusted threshold in the final model, demonstrating an effect size reduction of 6% overall and a final effect size of *β* = −0.080 (95% CI = −0.13 to −0.029, *P* = 2.33 × 10^−3^).

The turquoise module—enriched in several amino acid metabolism pathways—displayed negative associations with ACE-III, short-term memory and delayed memory (Model 1: *β* range = −0.12 to −0.09, *P* range = 5.23 × 10^−7^ to 2.05 × 10^−4^). All relationships remained at the nominal threshold in the final model, but none passed multiple testing correction (overall attenuation = 39.2–55.5%, *P* range = 0.0060–0.043). The majority of reductions came from Model 2 and 3 adjustments, with childhood cognition and education resulting in the largest attenuations (Supplementary Table 5).

Finally, we identified the brown module—enriched in diacylglycerol and phosphatidylethanolamine pathways—to be negatively associated with short-term memory at age 60–64 (Model 1: *β* = −0.075, 95% CI = −0.12 to −0.028, *P* = 1.85 × 10^−3^). Associations attenuated in Model 2, which appeared to be mainly driven by BMI (Supplementary Table 5). Subsequent model adjustments resulted in full attenuation.

#### Hub metabolites

**Figure 5:**
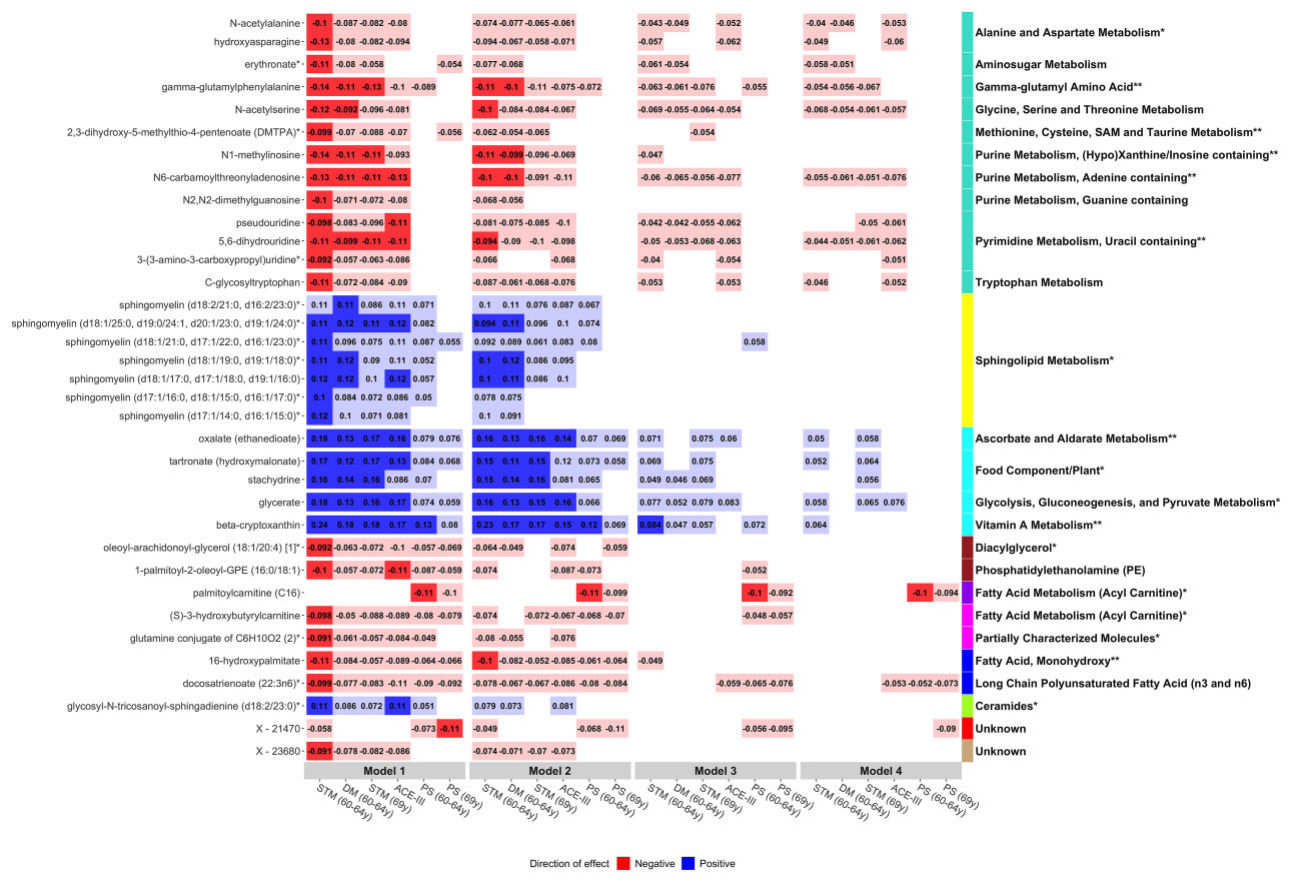
**Heatmap showing associations between hub metabolites and cognitive outcomes in Models 1–4**. Panels on the right indicate the pathways (text) and modules (colour) represented by metabolites. Pathways are suffixed with an asterisk if they were previously identified in our pathway analyses (**P < 9.43 × 10^−4^, *P < 0.05). Bonferroni-significant metabolites (*P* < 1.15 × 10^−4^) are represented by a solid fill, nominal metabolites by a faint fill (*P* < 0.05), and non-significant metabolites by no fill (*P* > 0.05). Tiles are coloured by effect direction and effect sizes are noted in the centre. For clarity purposes, only outcomes demonstrating a Bonferroni-significant result are shown. Source data are present in Supplementary Table 1. STM, short-term memory; PS, processing speed; DM, delayed memory; ACE-III, Addenbrooke’s Cognitive Examination-III.

Thirty-five metabolites identified in single-metabolite analyses were revealed to be hubs (kME > 0.65), and 28 of these belonged to the five modules that were associated with cognitive outcomes (Fig. 5 and Supplementary Table 1). Hubs further represented 8 of the 10 pathways identified in pathway analyses: gamma-glutamyl amino acid; methionine, cysteine, SAM and taurine metabolism; purine metabolism, (hypo)xanthine/inosine containing; purine metabolism, adenine containing; pyrimidine metabolism, uracil containing; ascorbate and aldarate metabolism; vitamin A metabolism and fatty acid (monohydroxy). In Model 4, one hub belonging to the purple module—palmitoylcarnitine (C16)—remained significant at the adjusted threshold and 17 were nominally significant. A correlation matrix of hub metabolites can be found in Supplementary Figure 5: correlations ranged from −0.35 to 0.91.

### Additional analyses

Due to sample size differences (above ACE-III threshold: *n* = 1190; *APOE*: max *n* = 1475; whole sample: *n* = 1740), results were examined for changes in significance from Bonferroni to non-significance (*P* > 0.05). For both single-metabolite and module analyses, additionally adjusting for *APOE* genotype did not change the pattern of results (Supplementary Table 6). Further exclusion of participants below the threshold for ACE-III resulted in changes to 15/155 metabolite associations, but all effect directions were preserved (highlighted in Supplementary Table 7). Of these, none were the four metabolites remaining in Model 4, but six were turquoise module hubs. For network analyses, associations were lost (*P* > 0.05) for the brown module and short-term memory (60–64 years), and the turquoise module and ACE-III, but no other changes were seen (Supplementary Table 7).

## Discussion

Using the British 1946 Birth Cohort, we systematically evaluated the metabolic correlates of cognitive function in late midlife while untangling influencing life course factors. Overall, we identified 155 metabolites, 10 pathways and 5 network modules to show associations with cognitive outcomes. Integrating these, 35 hub metabolites were revealed to show potential as markers for further study. Some of these relationships were independent of life course influences; however, consistent with our previous analyses in the MRC 1946,^6^ as well as a previous lipidomics study in the Lothian Birth Cohort,^38^ many were sensitive to childhood cognition and education, suggesting important considerations for future studies.

Most notably, we report independent relationships between the purple module—enriched in medium- and long-chain acylcarnitines—and processing speed, with our pathway analyses largely in support of this. These relationships were specific to processing speed, indicating a possible association unique to this outcome. One metabolite, palmitoylcarnitine (C16) (a long-chain acylcarnitine), appeared to be a key driver of these associations, suggesting a potential candidate for further investigation.

Biologically, medium- and long-chain acylcarnitines are derivatives of fatty acid metabolism and known to be pivotal in mitochondrial fatty acid oxidation.^39^ Increased abundances in serum have thus been regarded as proxies for mitochondrial dysfunction and impairments in subsequent energy production.^39^ Perturbations in acylcarnitine levels have been reported in early cognitive impairments and Alzheimer's disease,^7,40,41^ as well as other outcomes related to mitochondrial dysfunction, such as insulin resistance,^42,43^ obesity^43^ and cardiovascular disease.^44^ More specifically, palmitoylcarnitine has also been linked to the induction and regulation of apoptotic events.^45^ Both apoptosis and mitochondrial dysfunction have been implicated in neurodegeneration, indicating a plausible biological mechanism behind our observations.^46,47^ Future studies will seek to establish whether these changes lie on the causal pathway.

The turquoise module—comprised of nucleotides and amino acids—demonstrated negative associations with the ACE-III and memory outcomes; these were partly explained by life course factors, attenuating by 39.2–55.5% overall and nominally significant in the final model. Module hubs were amino acids and nucleosides that were unified by the presence of modifications, and included in these were several markers of RNA turnover and oxidative stress.^48–50^ Interestingly, many hubs have been reported together in a variety of outcomes, ranging from telomere length^50^ to chronic kidney disease.^51^ More recently, these metabolites were associated with a higher risk of multiple non-communicable diseases and all-cause mortality, with the top driver of the module—DMTPA (previously known as X-11564)—linked to eight different adverse outcomes.^52^ These widespread associations presents the possibility that they may reflect converging aetiologies, with a previous study hypothesizing that they may represent an ‘accelerated ageing’ phenotype.^53^

Next, a module enriched in vitamin A and C metabolites, cyan, showed positive associations with most cognitive outcomes, displaying the largest overall effects across all stages of analysis. Vitamin A and C metabolites are known antioxidants that may inhibit deleterious processes resulting from oxidative stress, which is thought to be involved in the pathogenesis of neurodegenerative diseases.^54,55^ Due to this, their involvement in ageing, cognitive decline and Alzheimer's disease has been discussed, with epidemiological studies showing conflicting results.^56,57^ Here, associations were sensitive to adjustment for life course factors, namely social factors and childhood cognition, providing a possible explanation for differing findings. Relationships were largely explained for processing speed and ACE-III in the final model, but remained at the nominal threshold for short-term memory.

Finally, we report relationships between sphingolipids and improved cognitive function which were entirely explained by life course factors, particularly childhood cognition and education. Sphingolipids are a lipid family comprised of sphingomyelins, ceramides and glycosphingolipids, and are present in large quantities in the CNS.^58^ Forming important components of cell membranes, they are highly dynamic and are thought to display essential roles in cognitive development and function.^59^ Supporting this, previous research has implicated disturbances in sphingolipid balance in cognitive development,^59,60^ function,^59^ ageing^59,61^ and Alzheimer's disease.^58^ Given these observational findings linking sphingolipids and cognitive function at several stages of the life course, unravelling the precise nature of these attenuations is warranted.

An important finding here is that many associations were attenuated after adjusting for social factors and childhood cognition. When following up on particular factors that may be driving this, we observed a large impact on childhood cognition and education, indicating that these factors may confound associations through influences on health-related traits that can, in turn, alter metabolite levels.^38^ Therefore, without prior adjustment for these measures, relationships with cognition later in life may be overestimated. Although many of our findings show attenuations, this does not necessarily indicate they are unimportant when looking at cognitive health and dementia risk; particularly for those that show biological relevance. In the case of relationships that show partial attenuations, this indicates possible lifelong bi-directional relationships between these metabolites and cognitive function, with education likely showing similar attenuation patterns via shared components. Nevertheless, with no earlier life metabolite data available, it is possible that later life metabolite levels may be a proxy for those in earlier life. As such, we cannot rule out the possibility that childhood cognition and education are influenced by prior metabolite levels. Alternatively, both metabolites and cognitive function could have shared genetic or environmental causes. When well-powered LC–MS GWAS are available, we will dissect the relationships highlighted here using MR to further understand potential cause and effect.

Findings should be considered in the light of several limitations. First, our results may be subject to residual confounding and a lack of longitudinal metabolomic data precludes the investigation into lifelong relationships and directionality. Next, cognitive change measures were curated from data collected within a narrow time window, which could explain the lack of relationships observed. Change measures were also represented by residualized change scores, which can be subject to bias, and our findings should be interpreted with this in caution. Finally, as seen with many cohort studies, individuals remaining in the study at this stage were generally of higher cognitive ability in childhood and more socially advantaged compared to the sample initially recruited at birth. Further, the study sample was ethnically homogenous. For generalization, it will be paramount to replicate this work in more diverse populations.

In summary, we conducted one of the largest LC–MS studies to date on cognitive outcomes in late midlife and are the first to evaluate systems-level associations in the context of life course factors. We integrated metabolites, pathways and networks, offering biological interpretation while retaining granularity, and highlighted molecular correlates of cognitive outcomes in late midlife. Our results illustrate the importance of incorporating life course influences, with many relationships largely explained by childhood cognition and education. Finally, we identified several metabolites (e.g. palmitoylcarnitine C16) that were both key in their module and associated with our outcomes, presenting as potential marker candidates for additional study.

## Supplementary Material

fcab291_Supplementary_Data

## Abbreviations

APOE: apolipoprotein E
ACE-III: Addenbrooke’s Cognitive Examination-III
BMI: body mass index
DASH: Dietary Approaches to Stop Hypertension
DMTPA: 2,3-dihydroxy-5-methylthio-4-pentenoic acid
GWAS: genome-wide association study
kME: module membership
LC–MS: liquid chromatography–mass spectrometry
MR: Mendelian randomisation
MRC: Medical Research Council
NSHD: National Survey of Health and Development
RNA: Ribonucleic acid
SEP: socioeconomic position
TOM: topological overlap matrix
WGCNA: weighted gene coexpression network analysis.
