# Supplementary material for: Metabolic correlates of late midlife cognitive outcomes: findings from the 1946 British Birth Cohort
Source: Brain Commun. 2021 Dec 15;4(1):fcab291. doi: 10.1093/braincomms/fcab291 (PMC8853724; doi:10.1093/braincomms/fcab291)
Supplement: fcab291_Supplementary_Data [file fcab291_supplementary_data.zip › Supplementary_material.docx]

Table of Contents

[Supplementary Tables 1](#_Toc86940366)

[Supplementary Methods 2](#_Toc86940367)

[Supplementary Figures 6](#_Toc86940368)

[Supplementary Figure 1. 7](#_Toc86940369)

[Supplementary Figure 2. 8](#_Toc86940370)

[Supplementary Figure 3 10](#_Toc86940371)

[Supplementary Figure 4 12](#_Toc86940372)

[Supplementary Figure 5 13](#_Toc86940373)

## Supplementary Tables

**Supplementary Table 1.** Full single-metabolite analyses results (models 1-4), including module membership and hub status.

**Supplementary Table 2.** Clinical and demographic characteristics of participants of the MRC 1946 British Birth Cohort study.

**Supplementary Table 3.** Linear regression analyses results for the association of each covariable against each predictor and outcome. All analyses are adjusted for sex, age at blood collection and blood collection centre.

**Supplementary Table 4.** Linear regression analyses results for the associations between modules and outcomes, after adjusting for each covariable individually.

**Supplementary Table 5.** Linear regression analyses results for the associations between metabolites and outcomes, after adjusting for each covariable individually. All analyses are adjusted for sex, age at blood collection and blood collection centre.

**Supplementary Table 6.** Linear regression analyses results for the associations between metabolites and modules vs outcomes, with and without adjusting for APOE. The maximum N with APOE genotype was used.

**Supplementary Table 7.** Linear regression analyses results for the associations between metabolites and modules vs outcomes, excluding individuals below the Addenbrooke’s Cognitive Examination-III clinical threshold (<82).

**Supplementary Table 8.** Pathway analyses results.

**Supplementary Table 9.** Module overrepresentation analysis results.

**Supplementary Table 10.** Full module analyses results (models 1-4).

## Supplementary Methods

Metabolomic profiling

*(Information provided by Metabolon Inc)*

**Sample Accessioning:** Following receipt, samples were inventoried and immediately stored at -80^o^C. Each sample received was accessioned into the Metabolon LIMS system and was assigned by the LIMS a unique identifier that was associated with the original source identifier only. This identifier was used to track all sample handling, tasks, results, etc. The samples (and all derived aliquots) were tracked by the LIMS system. All portions of any sample were automatically assigned their own unique identifiers by the LIMS when a new task was created; the relationship of these samples was also tracked. All samples were maintained at -80^o^C until processed.

**Sample Preparation:** Samples were prepared using the automated MicroLab STAR® system from Hamilton Company. Several recovery standards were added prior to the first step in the extraction process for QC purposes. To remove protein, dissociate small molecules bound to protein or trapped in the precipitated protein matrix, and to recover chemically diverse metabolites, proteins were precipitated with methanol under vigorous shaking for 2 min (Glen Mills GenoGrinder 2000) followed by centrifugation. The resulting extract was divided into five fractions: two for analysis by two separate reverse phase (RP)/UPLC-MS/MS methods with positive ion mode electrospray ionization (ESI), one for analysis by RP/UPLC-MS/MS with negative ion mode ESI, one for analysis by HILIC/UPLC-MS/MS with negative ion mode ESI, and one sample was reserved for backup. Samples were placed briefly on a TurboVap® (Zymark) to remove the organic solvent. The sample extracts were stored overnight under nitrogen before preparation for analysis.

**QA/QC:** Several types of controls were analyzed in concert with the experimental samples: a pooled matrix sample generated by taking a small volume of each experimental sample (or alternatively, use of a pool of well-characterized human plasma) served as a technical replicate throughout the data set; extracted water samples served as process blanks; and a cocktail of QC standards that were carefully chosen not to interfere with the measurement of endogenous compounds were spiked into every analyzed sample, allowed instrument performance monitoring and aided chromatographic alignment.Instrument variability was determined by calculating the median relative standard deviation (RSD) for the standards that were added to each sample prior to injection into the mass spectrometers. Overall process variability was determined by calculating the median RSD for all endogenous metabolites (i.e., non-instrument standards) present in 100% of the pooled matrix samples. Experimental samples were randomized across the platform run with QC samples spaced evenly among the injections.

**Ultrahigh Performance Liquid Chromatography-Tandem Mass Spectroscopy (UPLC-MS/MS):** All methods utilized a Waters ACQUITY ultra-performance liquid chromatography (UPLC) and a Thermo Scientific Q-Exactive high resolution/accurate mass spectrometer interfaced with a heated electrospray ionization (HESI-II) source and Orbitrap mass analyzer operated at 35,000 mass resolution. The sample extract was dried then reconstituted in solvents compatible to each of the four methods. Each reconstitution solvent contained a series of standards at fixed concentrations to ensure injection and chromatographic consistency. One aliquot was analyzed using acidic positive ion conditions, chromatographically optimized for more hydrophilic compounds. In this method, the extract was gradient eluted from a C18 column (Waters UPLC BEH C18-2.1x100 mm, 1.7 µm) using water and methanol, containing 0.05% perfluoropentanoic acid (PFPA) and 0.1% formic acid (FA). Another aliquot was also analyzed using acidic positive ion conditions, however it was chromatographically optimized for more hydrophobic compounds. In this method, the extract was gradient eluted from the same afore mentioned C18 column using methanol, acetonitrile, water, 0.05% PFPA and 0.01% FA and was operated at an overall higher organic content. Another aliquot was analyzed using basic negative ion optimized conditions using a separate dedicated C18 column. The basic extracts were gradient eluted from the column using methanol and water, however with 6.5mM Ammonium Bicarbonate at pH 8. The fourth aliquot was analyzed via negative ionization following elution from a HILIC column (Waters UPLC BEH Amide 2.1x150 mm, 1.7 µm) using a gradient consisting of water and acetonitrile with 10mM Ammonium Formate, pH 10.8. The MS analysis alternated between MS and data-dependent MS^n^ scans using dynamic exclusion. The scan range varied slighted between methods but covered 70-1000 m/z. Raw data files are archived and extracted as described below.

**Data Extraction and Compound Identification:** Raw data was extracted, peak-identified and QC processed using Metabolon’s hardware and software. These systems are built on a web-service platform utilizing Microsoft’s .NET technologies, which run on high-performance application servers and fiber-channel storage arrays in clusters to provide active failover and load-balancing. Compounds were identified by comparison to library entries of purified standards or recurrent unknown entities. Metabolon maintains a library based on authenticated standards that contains the retention time/index (RI), mass to charge ratio (*m/z)*, and chromatographic data (including MS/MS spectral data) on all molecules present in the library. Furthermore, biochemical identifications are based on three criteria: retention index within a narrow RI window of the proposed identification, accurate mass match to the library +/- 10 ppm, and the MS/MS forward and reverse scores between the experimental data and authentic standards. The MS/MS scores are based on a comparison of the ions present in the experimental spectrum to the ions present in the library spectrum. While there may be similarities between these molecules based on one of these factors, the use of all three data points can be utilized to distinguish and differentiate biochemicals. More than 3300 commercially available purified standard compounds have been acquired and registered into LIMS for analysis on all platforms for determination of their analytical characteristics. Additional mass spectral entries have been created for structurally unnamed biochemicals, which have been identified by virtue of their recurrent nature (both chromatographic and mass spectral). These compounds have the potential to be identified by future acquisition of a matching purified standard or by classical structural analysis.

**Curation:** A variety of curation procedures were carried out to ensure that a high quality data set was made available for statistical analysis and data interpretation. The QC and curation processes were designed to ensure accurate and consistent identification of true chemical entities, and to remove those representing system artifacts, mis-assignments, and background noise. Metabolon data analysts use proprietary visualization and interpretation software to confirm the consistency of peak identification among the various samples. Library matches for each compound were checked for each sample and corrected if necessary.

**Metabolite Quantification and Data Normalization:** Peaks were quantified using area-under-the-curve. A data normalization step was performed to correct variation resulting from instrument inter-day tuning differences. Essentially, each compound was corrected in run-day blocks by registering the medians to equal one (1.00) and normalizing each data point proportionately.

## Supplementary Figures

Supplementary Figure 1. **Flow chart depicting metabolomic data quality control.**

Supplementary Figure 2. **Scale free topology and mean connectivity plots for weighted gene coexpression network analysis.**

Supplementary Figure 3 **(previous page). Upset plot depicting the number of metabolites associated with each outcome and combination of outcomes at the adjusted threshold, split by metabolite family.** Outcomes are shown in the matrix, with shaded circles demonstrating those represented by the bar chart above. Where more than one outcome is indicated, lines further highlight these intersections. Bars are coloured by metabolite family and total metabolite counts are displayed on top of each bar, alongside the corresponding percentage proportion of all significant metabolites identified across all outcomes. As no metabolites were significant at the adjusted threshold for change outcomes, these were not included. (Left) Barplot showing the number of metabolites identified overall for each outcome. Bars are coloured by metabolite family and counts are displayed on the top of each bar. Source data are present Supplementary Table 1.

Abbreviations: *ACE-III = Addenbrooke’s Cognitive Examination-III.*

**
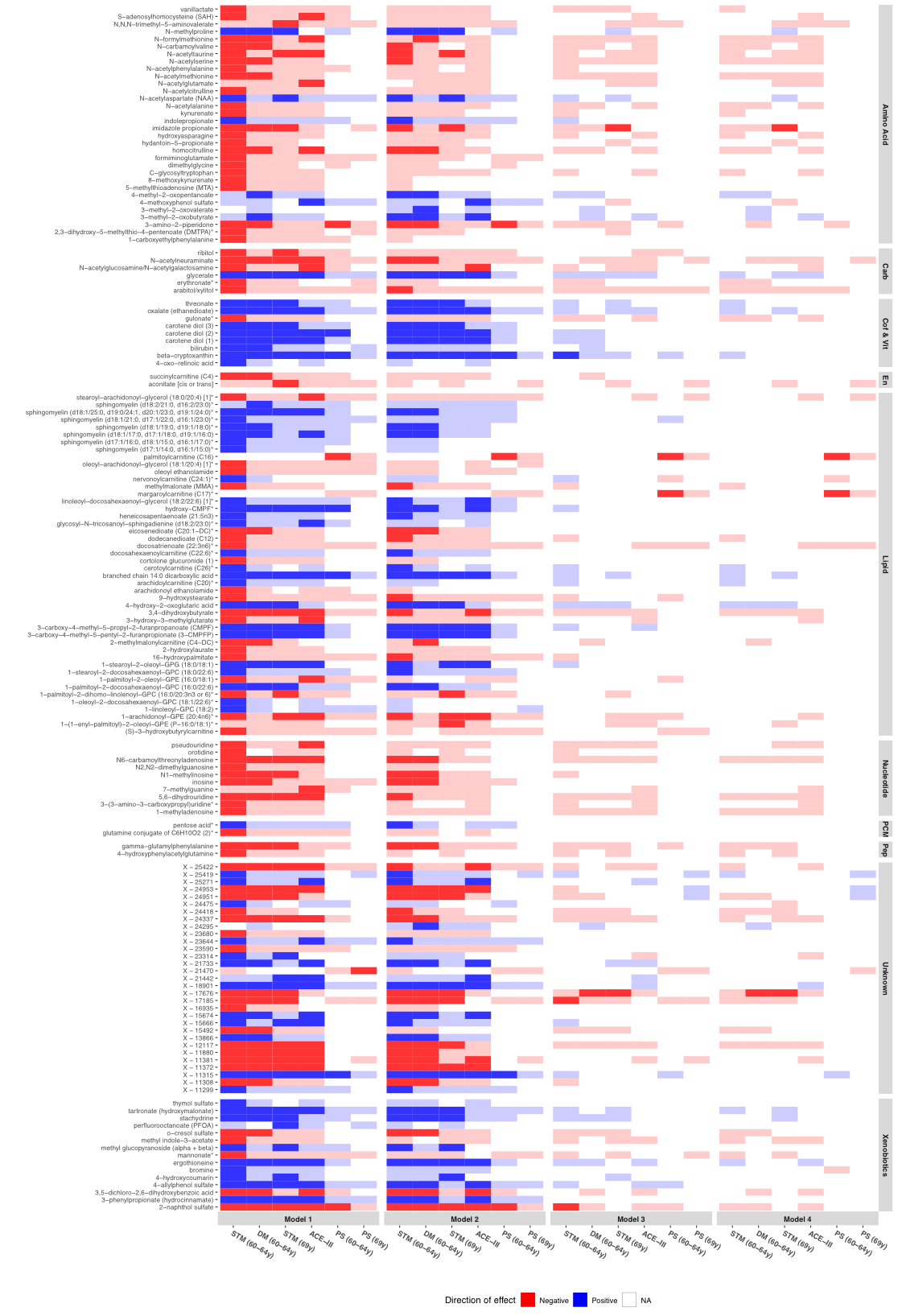
**

Supplementary Figure 4 **(previous page). Heat map showing trends of associations between the 155 metabolites and cognitive outcomes in models 1-4, organised by metabolite family.** Bonferroni-significant metabolites (p<1.15x10^-4^) are represented by a solid fill, nominal metabolites by a faint fill (p<0.05), and non-significant metabolites by no fill (p>0.05).

Abbreviations: *Carb = carbohydrates, Cof & Vit = cofactors & vitamins, En = energy, PCM = partially characterised molecules, Pep = peptides, ACE-III = Addenbrooke’s Cognitive Examination-III, DM = delayed memory, STM = short-term memory, PS = processing speed*

Supplementary Figure 5**. Correlation matrix showing relationships between hub metabolites.**

Metabolite names are indicated and colour panels on the top and right-hand side of the

matrix indicate the modules represented by metabolites. Correlations are indicated inside

the tile.
